# Determination of Efficiency of 3D Fluid-Attenuated Inversion Recovery (FLAIR) in the Imaging of Multiple Sclerosis in Comparison With 2D FLAIR at 3-Tesla MRI

**DOI:** 10.7759/cureus.48136

**Published:** 2023-11-01

**Authors:** Satyanarayana Kummari, Kiran Goud Burra, Vudem Ranjith Kumar Reddy, Saraswata Das, Challa Anilkumar

**Affiliations:** 1 Department of Radiology, Great Eastern Medical School & Hospital, Srikakulam, IND; 2 Department of Radiology, Government District Hospital, Medak, Medak, IND; 3 Department of Radiology, Surabhi Institute of Medical Sciences, Siddipet, IND; 4 Department of Radiodiagnosis, College of Medicine and JNM Hospital, Kalyani, IND

**Keywords:** 3-tesla mri, cnr, snr, comparison, mri, 3d-flair, 2d-flair, multiple sclerosis

## Abstract

Background: A fluid-attenuated inversion recovery (FLAIR) method eliminates the cerebrospinal fluid (CSF) signal, enhancing white matter lesion detection by enhancing the contrast between the lesion and CSF. Three-dimensional (3D) volume acquisition has the advantage of multiplanar reformation of contiguous slices yielding improved signal-to-noise ratios (SNRs) and contrast-to-noise ratios (CNRs). To our knowledge, there are only three studies comparing 3D- and 2D-FLAIR sequences with respect to multiple sclerosis (MS) lesions at 3 tesla.

Aims and objectives: This study aimed to determine the efficiency of 3D-FLAIR in the detection of lesions of multiple sclerosis in terms of spatial and contrast resolutions in comparison with 2D-FLAIR sequences.

Methodology: A total of 75 patients with MS undergoing magnetic resonance imaging (MRI) brain at the Department of Radiology, Krishna Institute of Medical Sciences (KIMS), Secunderabad, Telangana, India. This is an observational comparative study. Independent-samples t-tests were performed in the present study to compare the number of lesions detected. The measured CNR and SNR values were subjected to Mann-Whitney U test.

Results: As a result of the 3D-FLAIR, more lesions were found as compared to 2D-FLAIR (p = 0.001). There was a greater CNRs for 3D-FLAIR images than for 2D-FLAIR images (p = 0.001). Lesions, CSF, white matter, and gray matter showed significantly higher SNRs with 3D-FLAIR (p = 0.001).

Conclusion: 3D-FLAIR has exhibited greater sensitivity in detecting lesions associated with MS when contrasted with the 2D-FLAIR sequence. Significantly more lesions and higher SNRs and CNRs were detected with 3D-FLAIR in contrast to 2D-FLAIR. 3D-FLAIR may be considered the sequence of choice for MS imaging in the future.

## Introduction

Magnetic resonance imaging (MRI) plays a chief role in the diagnostic workup of multiple sclerosis (MS) [1,2]. Hence, MRI was a key component in both the original 2001 McDonald criteria and their 2005, 2010, and 2017 revised versions [3,4]. Fluid-attenuated inversion recovery (FLAIR) merges a high intensity of T2 weighting with the attenuation of cerebrospinal fluid (CSF) signal [5,6]. In this study, FLAIR was primarily performed as a 2D acquisition. Subsequently, newer 3D sequences have been implemented. These 3D sequences allow thin contiguous slices with multiplanar reconstruction, higher spatial resolution, good signal-to-noise ratios (SNRs) and contrast-to-noise ratios (CNRs), and thereby improved detection of smaller lesions [7].

Initially, 3D-FLAIR sequences were primarily conducted using a multislab approach, which led to the emergence of Venetian blind artifacts when the resulting images were remodeled [8]. The single slab type of the 3D-FLAIR sequence was taken into consideration to eliminate this artifact [9]. The field strength is directly proportional to a high SNR. The enhanced SNR observed at higher field strengths in imaging can be harnessed either to shorten the imaging duration or enhance spatial resolution [10]. Markedly reduced motion artifacts were seen with a single-slab 3D-FLAIR sequence as compared to a 2D-FLAIR sequence in brain imaging at 1.5 Tesla (T) [11].

Furthermore, almost no CSF flow artifacts were seen with the 3D-FLAIR sequence in brain imaging [12]. In addition, 3D datasets are compatible with computer-aided analysis [13]. The present study aims to test the single-slab 3D-FLAIR sequence for MS at 3 T. For this purpose, we collated a single-slab 3D-FLAIR sequence to a standard 2D-FLAIR sequence considering the number of MS lesions detected, SNRs, and CNRs in the brain.

## Materials and methods

This is an observational comparative study, and an informed consent form was obtained from all the included participants. The Institutional Human Ethics Committee (IHEC) of KIMSHEALTH and Dr. P. Raja Gopal, Director of Medical Education, issued approval (approval date: March 15, 2016). Seventy-five patients (18 males and 57 females, between 5 and 65 years) with clinically definite MS undergoing MRI brain at the Department of Radiology, Krishna Institute of Medical Sciences (KIMS), Secunderabad, Telangana, India, were included in this study. 

Inclusion and exclusion criteria

All the patients with clinically diagnosed MS undergoing MRI brain imaging at the department of radiology were included in the study.

Patients with concomitant neurological disease in addition to MS; patients with a history of claustrophobia; patients with cardiac pacemakers, prosthetic heart valves, cochlear implants, or any metallic implants in the body; and patients who did not consent to be a part of the study were excluded from the study.

MR image acquisition

A 16-channel phased-array HR SENSE NV 16 coil was used on a 3T MRI system (Philips Achieva TX, Netherlands). We have obtained an additional sequence (3D-FLAIR) along with routine MRI sequences (including 2D-FLAIR) for MS. 3D-FLAIR refers to single-slab 3D fast spin echo (FSE) T2 FLAIR, which is an advanced MRI sequence. Very long spin-echo trains are created by utilizing variable flip-angle refocusing radiofrequency (RF) pulses. As a result, this approach gives a slower signal decay when compared to traditional FSE sequences that employ a continuous flip angle of 180° (13). In addition, employing refocusing RF pulses with flip angles substantially below 180° can result in a reduction in the specific absorption rate (SAR).

The 2D-FLAIR sequence was acquired in an axial plan, while the 3D-FLAIR sequence was obtained in the sagittal plane, which was then reformatted into axial slices. The width of slices of 2D-FLAIR and 3D-FLAIR was 5 mm and 0.9 mm, respectively. Imaging parameters used for the 2D- and 3D-FLAIR sequences were as follows: (a) repetition time msec/echo time msec, 10000/125; inversion time msec, 2800 and matrix, 300x205 for the 2D-FLAIR sequence (the remaining parameters are as follows: 5 mm section thickness with a 2 mm intersection gap and 230x230x142 mm field of view); (b) repetition time msec/echo time msec, 4800/315; inversion time msec, 1650 and matrix, 252x249 for the 3D-FLAIR sequence (the other variables were as follows: 0.9 mm section thickness with no intersection gap and 250x250x168 mm field of view).

Image analysis

**Figure 1:**
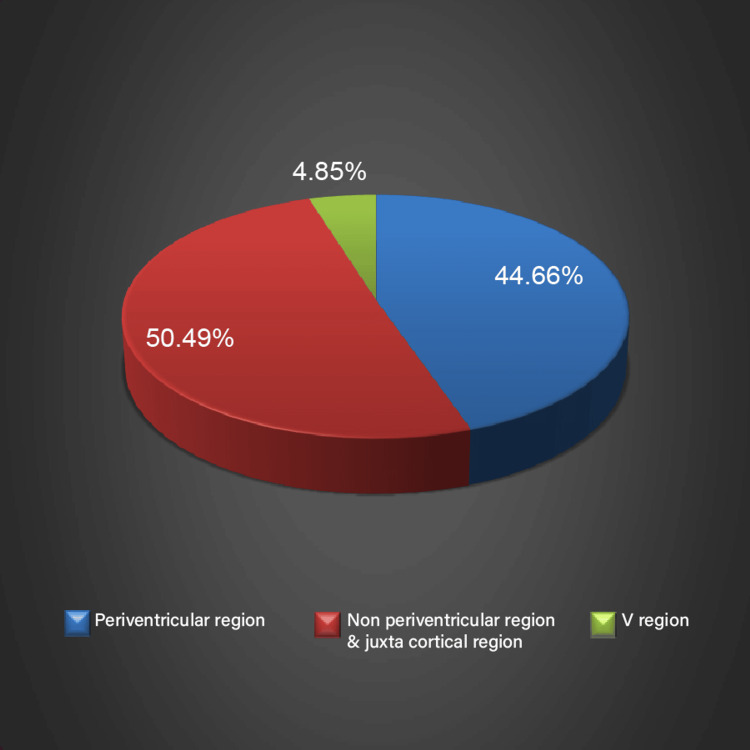
Distribution of lesions on 2D-FLAIR

**Figure 2:**
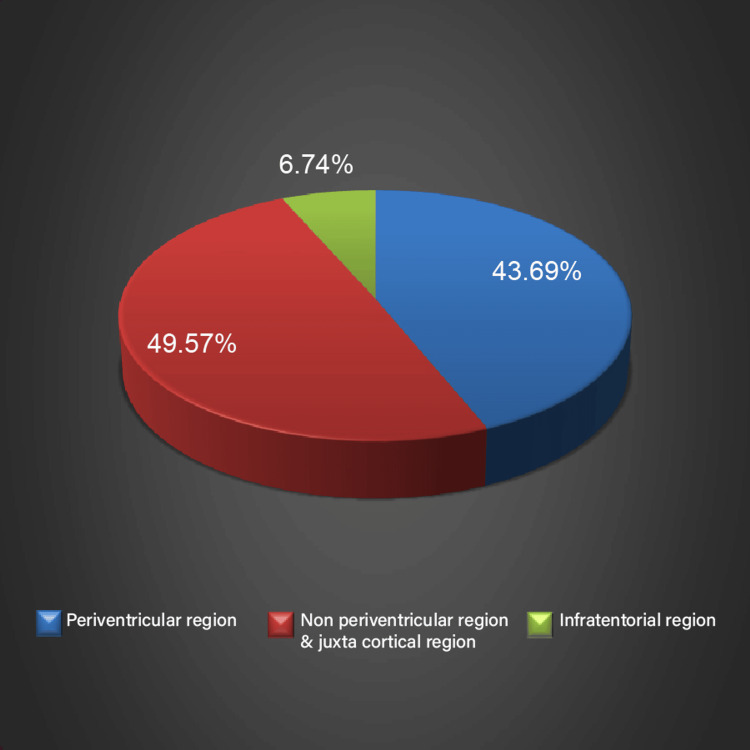
Distribution of lesions on 3D-FLAIR

Only axial slices were used for the evaluation. Two neuroradiologists, each possessing a minimum of five years of experience in the field, assessed the lesions and reviewed the sequences in a randomized manner. Lesions were calculated and assigned to the following brain regions: periventricular (PV), non-periventricular/juxtacortical (NPV & JC), and infratentorial (IT) (Figure 1 and Figure 2). The analysis of CNR was done through a random selection of three lesions from three regions (PV, NPV & JC, and IT) one from each region in each patient. In each patient, the same three lesions were assessed for signal intensities (SIs) in both types of sequences used. The CNR was measured as SI lesion-SI tissue/Std noise, and SNR was calculated as SI tissue/Std noise. To prevent any artifacts, the standard deviation of the noise was assessed in a designated area for study outside of the brain.

Statistical analysis

In this study, we used IBM SPSS Statistics for Windows, version 17 (released 2008; IBM Corp., Armonk, New York, United States) to perform all statistical calculations. The total number of lesions identified on two sequences was compared by using an independent sample t-test. The CNRs and SNRs measured on the two sequences were calculated using a Mann-Whitney U test.

## Results

Table 1 demonstrates the number of lesions detected in 75 patients. As compared to 2D-FLAIR, 3D-FLAIR detected significantly more lesions (5,378; p-value = 0.001). In comparison to 2D-FLAIR images, the 3D-FLAIR images exhibited significantly elevated SNRs for lesions, CSF, white matter, and gray matter and markedly increased CNR intermediating lesions and all the three above-mentioned reference tissues with a p-value of 0.001 (Table 2).

**Table 1:** Number of lesions detected by two-dimensional and three-dimensional FLAIR sequences. FLAIR: fluid-attenuated inversion recovery

	2D-FLAIR	3D-FLAIR	p-value
Periventricular region	1481	2350	0.001
Non-periventricular region and juxta-cortical region	1675	2665	0.001
Infratentorial region	161	363	0.001

**Table 2:** (Mean ± standard deviation) contrast-to-noise ratio (CNR) and signal-to-noise ratio (SNR) values. GM: gray matter; WM: white matter; CSF: cerebrospinal fluid; SNR: signal-to-noise ratio; CNR: contrast-to-noise ratio; FLAIR: fluid-attenuated inversion recovery; SD: standard deviation

		2D-FLAIR		3D-FLAIR		p-value
		Mean	SD	Mean	SD	
SNR	Lesion	423.20	362.14	786.02	536.72	0.001
	CSF	30.10	23.98	43.85	31.37	0.001
	WM	254.08	206.55	385.57	284.49	0.001
	GM	329.53	235.48	534.08	405.21	0.001
CNR lesion	CSF	387.11	341.91	621.06	526.76	0.001
	WM	229.70	224.38	334.38	309.01	0.001
	GM	144.82	138.47	198.37	174.15	0.001

## Discussion

When the 3D-FLAIR and 2D-FLAIR sequences were compared, the single-slab 3D-FLAIR sequence was dominant over the 2D-FLAIR sequence regarding the evaluation of the number of lesions and higher SNRs and CNRs. To our knowledge, there are only three studies comparing 3D- and 2D-FLAIR sequences regarding MS lesions at 3T MRI. 2D-FLAIR has a greater potential for lesion detection than traditional 2D spin echo sequences, as demonstrated in the literature [5,14-18]. Multislab implementation served as the foundation for the first 3D-FLAIR experience. Despite the extended acquisition times associated with multislab 3D-FLAIR, the clear advantages in terms of enhanced lesion detection, improved uniformity in CSF suppression, a heightened SNR per unit of time, narrow image slices, and the ability to reformulate images in any desired plane were evident [19]. However, undesirable magnetization transfer phenomena and slab boundary artifacts are encountered in the multislab mode [20,21]. In the year 2000, a single-slab 3D-FLAIR sequence was introduced, effectively eliminating the magnetization transfer phenomena and slab boundary artifacts while allowing simultaneously thin contiguous slices to elevate the anatomical details and detection of smaller lesions. The spatial resolution is higher with single-slab 3D-FLAIR sequences than with conventional 2D-FLAIR sequences [9,11].

The subtype of the 3D FSE T2 FLAIR sequence is the 3D-FLAIR sequence. Utilizing modulated flip-angle refocusing RF pulses enables the generation of extended echo trains, primarily because they yield a more gradual signal decay owing to their high output. In this way, more echoes are generated without causing extensive blurring and artifacts [8]. In addition, 3D sequence flip angles are commonly less than 180°, thus resulting in a low specific absorption rate.

**Figure 3:**
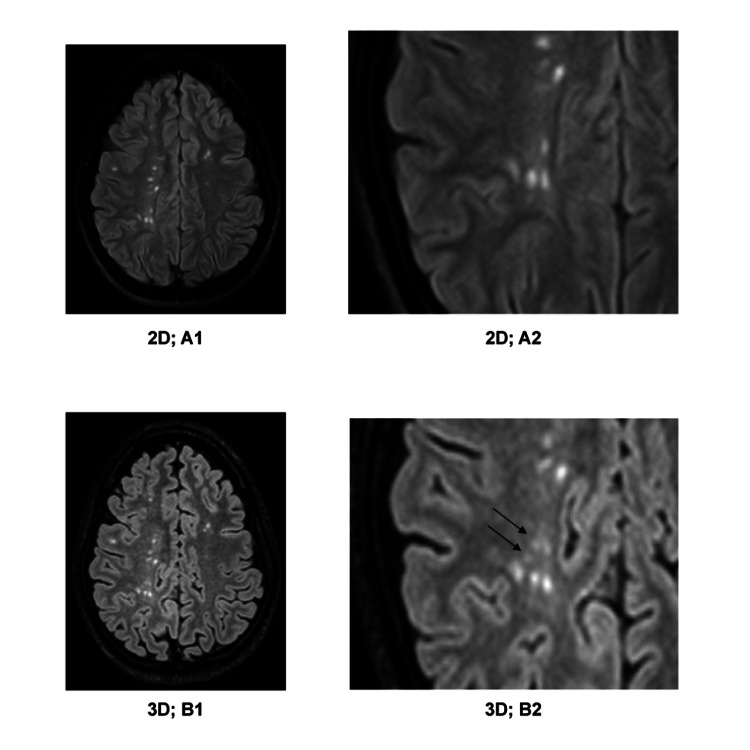
A1) 2D-FLAIR image; A2) detail enlargements of A1; B1) 3D-FLAIR image, approximately corresponding to A1; B2) detailed enlargement of B1. On the 3D-FLAIR images, white matter lesions in the right centrum semiovale (marked with arrows in B2) are visible, which cannot be detected on the 2D-FLAIR images. White matter lesions can be more clearly distinguished on the 3D-FLAIR image.

**Figure 4:**
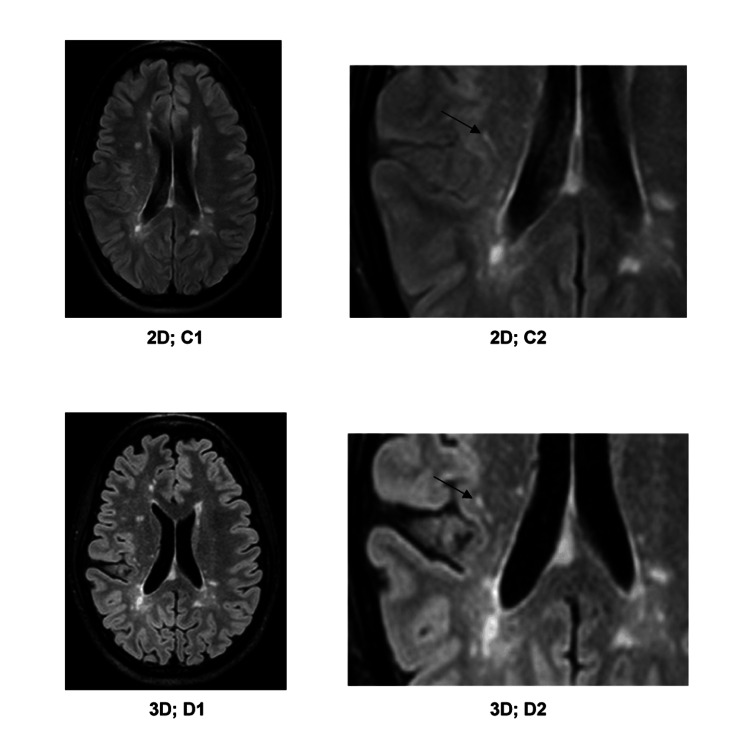
C1) 2D-FLAIR image; C2) detailed enlargement of C1; D1) 3D-FLAIR image, roughly aligning with C1; D2) detailed enlargement of D1. The right parietal lesion (marked with an arrow in C2) actually consists of two separate lesions (marked with an arrow in D2). White matter lesions exhibit clearer differentiation on the 3D-FLAIR image.

**Figure 5:**
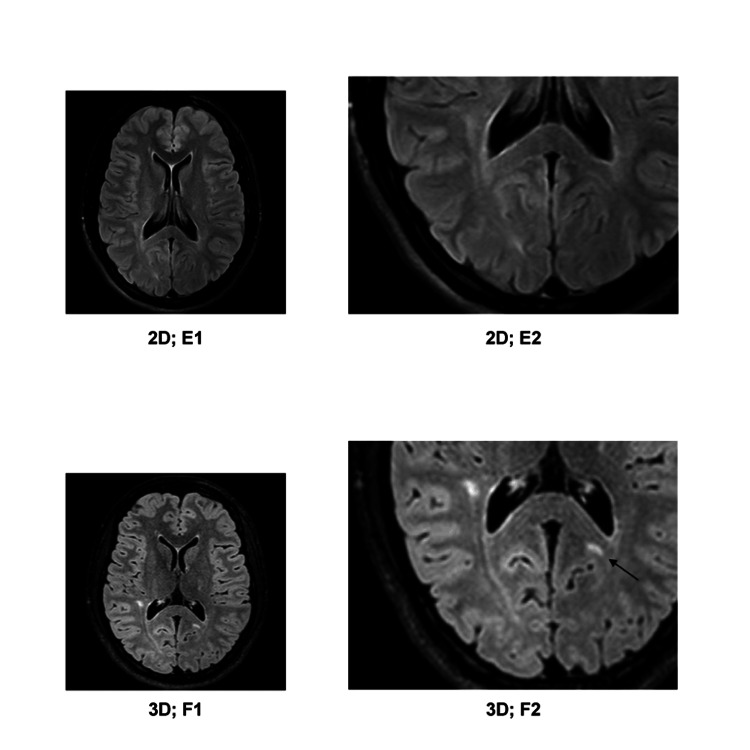
E1) 2D-FLAIR image; E2) detailed enlargement of E1; F1) 3D-FLAIR image, roughly aligning to E1; F2) detailed enlargement of F1. The lesion in the splenium of corpus callosum (marked with an arrow in F2) is visible on 3D-FLAIR images, which remain undetected on the 2D-FLAIR images.

**Figure 6:**
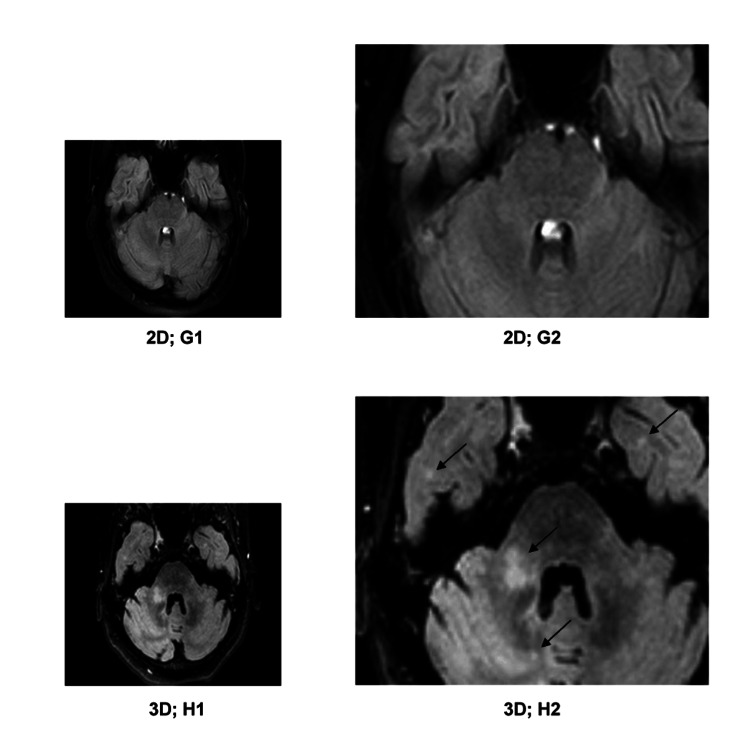
G1) 2D-FLAIR image; G2) detailed enlargement of G1; H1) 3D-FLAIR image, approximately corresponding to G1; H2) detailed enlargement of H1. On the 3D-FLAIR images, white matter lesions in the right middle cerebellar peduncle and bilateral temporal lobes (marked with arrows in H2) are visible, which remained undetected on the 2D-FLAIR images.

**Figure 7:**
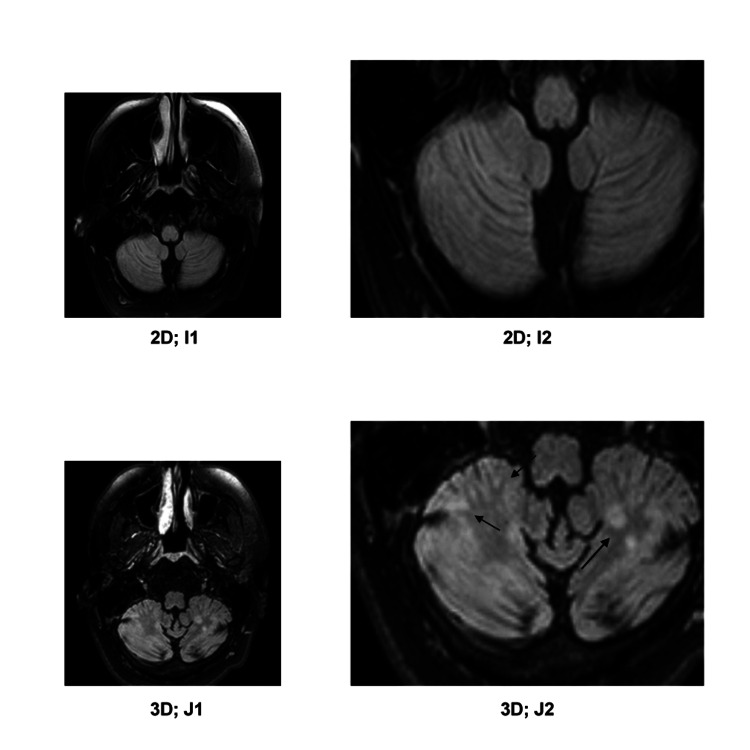
I1) 2D-FLAIR image; I2) detailed enlargement of I1; J1) 3D-FLAIR image, roughly aligning with I1; J2) detailed enlargement of J1. On the 3D-FLAIR images, six lesions in the bilateral-cerebellar hemispheres (marked with arrows in J2) are visible, which are undetected on the 2D-FLAIR images.

3D-FLAIR shows more lesions when 2D-FLAIR and 3D-FLAIR sequences are compared. This was due to the identification of small lesions that remained undetected on 2D-FLAIR images (Figure 3, Figures 5-7). Moreover, it was possible to distinctly appreciate the discrete lesions apparently confluent on the 2D-FLAIR sequence (Figure 4). Multislab 3D-FLAIR acquisition with decreasing slice thickness (5, 3, and 1 mm) demonstrated improved detection of small lesions at 1.5T [22]. Our results specifically highlighted the impact of slice thickness, comparing 0.9 mm slices with no inter-slice gap to 5 mm slices with a 2.2 mm inter-slice gap. The 3D-FLAIR sequence revealed a total of 2,061 additional lesions compared to the 2D-FLAIR sequence (Table 1). The interslice gap within the 2D-FLAIR sequence is a contributing factor to the reduced detection of lesions. In our study, we observed that the number of detected lesions in the infratentorial region was notably limited, a finding consistent with the observations of other researchers. For this reason, McGowan and Patel [23] suggested that the FLAIR sequence was not recommended for the infratentorial region due to the significantly distinct relaxation times compared to the supratentorial parenchyma. While 3D-FLAIR may increase the identification of supratentorial white matter lesions, alternative sequences may be required for the best imaging of the infratentorial regions.

3D sequences offer advantages in terms of signal strength and contrast properties, even when utilizing small voxels [7,24,25]. Our measurements confirm these results, demonstrating higher values for all calculated ratios. The assessment of SNR and CNR values followed a methodology like the one outlined by Tan et al. [8]. In the 3D-FLAIR images, the SNR values were significantly higher than those in the 2D-FLAIR images (p-value = 0.001) for lesions, CSF, white matter, and gray matter. In the present study, we compared 3D-FLAIR and 2D-FLAIR images; our results show that the SNR values for lesions, CSF, white matter, and gray matter were notably higher in the 3D-FLAIR images, with a very low p-value of 0.001. Therefore, 3D-FLAIR imaging gives better image quality and signal clarity for these brain structures compared to 2D-FLAIR imaging (Table 2). A 2D-FLAIR sequence used a conventional spin echo train with 180° refocusing RF pulses, while a 3D-FLAIR sequence used an extended spin echo train with different flip angles. The above-mentioned difference in the echo trains could reasonably result in quantitative disparities in signal intensities.

In the present investigation, three reference tissues (CSF, white matter, and gray matter) showed significantly higher CNR values in 3D-FLAIR images (p-value = 0.001) than in 2D-FLAIR images (Table 2). This improvement can be attributed to the single-slab design inherent to the 3D-FLAIR sequence. The 2D-FLAIR sequence is acquired in four minutes. In addition, the 3D-FLAIR scan can be performed in five minutes, which is clinically acceptable. We hold the view that employing a standard diagnostic protocol comprising 3D-FLAIR, a T2-weighted sequence specifically tailored for infratentorial imaging, and a 3D T1-weighted sequence with pre- and post-gadolinium contrast administration offers a high degree of sensitivity and effectiveness in MS imaging.

Our study has a few limitations. A limited number of patients were examined without the inclusion of a control group. 2D-FLAIR sequence with a slice thickness of 5 mm and interslice gap of 2.2 mm differed substantially from the single-slab 3D-FLAIR sequence with a slice thickness of 0.9 mm and no interslice gap. Optimal imaging of the infratentorial regions requires a variety of sequences because FLAIR sequences may not be effective for this area. We did not assess the potential utility of 3D-FLAIR imaging in detecting lesions in the spinal cord, cortical regions, and optic nerves. Cortical and optic nerve evaluations were introduced into the 2016 Magnetic Resonance Imaging in Multiple Sclerosis (MAGNIMS). It is plausible that improvements in MRI techniques will influence the diagnostic criteria for MS. The latest additions to the McDonald criteria, incorporating the MR criteria, have relied on imaging studies conducted at a magnetic field strength of 1.5T. The combination of 3T MRI with 3D-FLAIR sequence, as shown in our study, can be valuable in diagnosing MS.

## Conclusions

A single-slab 3D-FLAIR sequence identifies a greater number of lesions as compared to a 2D-FLAIR sequence. The upgraded presentation of 3D-FLAIR sequences can be recognized to the presence of a substantial quantity of thin, contiguous slices, high spatial resolution, and remarkable increase in the SNRs and CNRs provided by the technique. The effectiveness of 3D sequences in MS diagnosis is still under research and their potential. However, the confirmation of this will require larger-scale studies. The study indicates that 3D-FLAIR is superior to the conventional 2D-FLAIR with respect to MS imaging. 3D-FLAIR can take over 2D-FLAIR in MS imaging in the future. Diagnosis of MS at the initial phase can be expected by using high-field MRI combined with 3D sequences.
